# An ACE2-dependent Sarbecovirus in Russian bats is resistant to SARS-CoV-2 vaccines

**DOI:** 10.1371/journal.ppat.1010828

**Published:** 2022-09-22

**Authors:** Stephanie N. Seifert, Shuangyi Bai, Stephen Fawcett, Elizabeth B. Norton, Kevin J. Zwezdaryk, James Robinson, Bronwyn Gunn, Michael Letko

**Affiliations:** 1 Paul G. Allen School for Global Health, Washington State University, Pullman, Washington, United States of America; 2 Department of Microbiology and Immunology, Tulane University School of Medicine, New Orleans, Louisiana, United States of America; 3 Department of Pediatrics, Tulane University School of Medicine, New Orleans, Louisiana, United States of America; Colorado State University College of Veterinary Medicine and Biomedical Sciences, UNITED STATES

## Abstract

Spillover of sarbecoviruses from animals to humans has resulted in outbreaks of severe acute respiratory syndrome SARS-CoVs and the ongoing COVID-19 pandemic. Efforts to identify the origins of SARS-CoV-1 and -2 has resulted in the discovery of numerous animal sarbecoviruses–the majority of which are only distantly related to known human pathogens and do not infect human cells. The receptor binding domain (RBD) on sarbecoviruses engages receptor molecules on the host cell and mediates cell invasion. Here, we tested the receptor tropism and serological cross reactivity for RBDs from two sarbecoviruses found in Russian horseshoe bats. While these two viruses are in a viral lineage distinct from SARS-CoV-1 and -2, the RBD from one virus, Khosta 2, was capable of using human ACE2 to facilitate cell entry. Viral pseudotypes with a recombinant, SARS-CoV-2 spike encoding for the Khosta 2 RBD were resistant to both SARS-CoV-2 monoclonal antibodies and serum from individuals vaccinated for SARS-CoV-2. Our findings further demonstrate that sarbecoviruses circulating in wildlife outside of Asia also pose a threat to global health and ongoing vaccine campaigns against SARS-CoV-2

## Author summary

SARS-CoV-2, the sarbecovirus behind COVID-19, emerged in the human population after cross-species transmission from an animal source. While hundreds of sarbecoviruses have been discovered, predominantly in bats in Asia, the majority are not capable of infecting human cells. Khosta-2, a sarbecovirus discovered in Russia, has been shown to interact with the same entry receptor as SARS-CoV-2. In this study, we tested how well the spike proteins from these bat viruses infect human cells under different conditions. We found that the spike from virus, Khosta-2, could infect cells similar to human pathogens using the same entry mechanisms, but was resistant to neutralization by serum from individuals who had been vaccinated for SARS-CoV-2.

## Introduction

Zoonotic spillover of sarbecoviruses from animals to humans has led to the emergence of highly pathogenic human viruses, SARS-CoV-1 and -2, with the later leading to the largest global pandemic in modern history. Researchers around the world are ramping up the pace of viral discovery efforts, expanding the sequence databases with new animal sarbecoviruses in circulation. While some laboratory experiments have been performed with these new viruses, demonstrating a range of host tropisms, several viruses remain untested, and thus their ability to transmit to humans is unknown.

Coronaviruses are covered with a spike glycoprotein (S) that engages with receptor molecules on the surface of host cells and mediates viral infection of the cell. A small region within the spike proteins of sarbecoviruses, known as the receptor binding domain (RBD), contains all of the structural information necessary to engage with the host receptor. We and others have experimentally classified the majority of published sarbecovirus RBDs into different clades based on sequence and functional data: clade 1, identified in Asian bats, contains no deletions and binds to host receptor, Angiotensin-Converting Enzyme 2 (ACE2), whereas clade 2, also identified in Asian bats, contains 2 deletions and does not use ACE2 and clade 3 viruses, found more widely in African and European bats, contain 1 deletion and have recently been shown can infect using primarily bat ACE2 [1–10]. In 2021, several viruses were identified in China that comprise a fourth clade that also interact with ACE2 [11].

In late 2020, two clade 3 sarbecoviruses were identified in Rhinolophus bats in Russia: Khosta-1 was found in *Rhinolophus ferrumequinum* and Khosta 2 in *R. hipposideros [12]*. Similar to other European and African clade 3 viruses, the Khosta viruses are divergent from the RBD found in SARS-CoV-1 and -2. Here, we confirm ACE2 receptor preference in these and other clade 3 viruses using pseudotyped virus-like particles with both chimeric and full-length clade 3 spikes. We also assessed the antibody neutralization of a chimeric SARS-CoV-2 spike encoding for the RBD from Khosta 2 virus to assess the protection offered by current SARS-CoV-2 vaccines against future sarbecovirus threats. Critically, our findings highlight the urgent need to continue development of new, and broader-protecting sarbecovirus vaccines.

## Results

### Khosta virus receptor binding domains are distinct from human viruses

**Fig 1:**
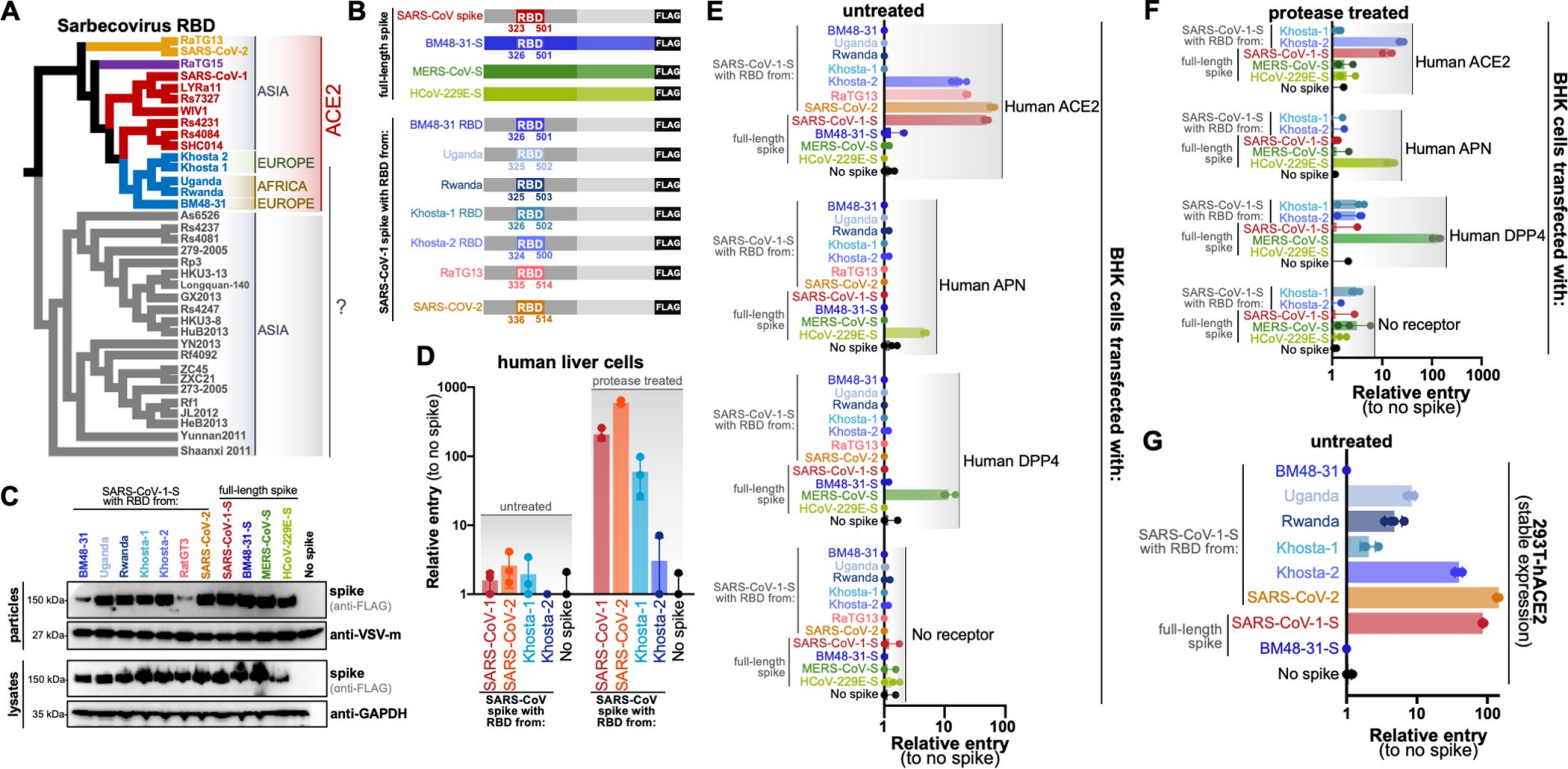
Khosta 2 and other RBD clade 3 sarbecoviruses use human ACE2 to infect cells. (**A**) Sarbecovirus Receptor Binding Domain Cladogram based on amino acid sequences and rooted at the midpoint. Countries of origin and known host receptors are indicated to the right. Clade 1 viruses are shown in red and orange, clade 2 in grey, clade 3 in blue and clade 4 in purple. (**B**) Diagram of spike constructs used for this study. The SARS-CoV-1 RBD was replaced with RBDs from other sarbecoviruses. (**C**) Expression and incorporation of viral pseudotypes by westernblot. (**D**) Huh-7 cells were infected with pseudotypes in the presence of absence of trypsin. Cells were infected in triplicate. (**E**) BHK cells were transfected with receptors and infected in the absence (**F**) or presence of trypsin. (**G**) 293T cells stably over-expressing human ACE2 were infected with pseudotypes without trypsin. Cells were infected in triplicate or quadruplicate for experiments in D-G.

Khosta-1 and -2 were identified by Alkhovsky and colleagues in bat samples collected between March-October 2020 near Sochi National Park [12]. Phylogenetic analysis of the conserved viral gene, Orf1ab, revealed these viruses were most closely related to another sarbecovirus found in Bulgaria in 2008 (known as BM48-31 or Bg08), and form a lineage sarbecoviruses distinct from human pathogens, SARS-CoV-1 and -2 [12]. A list of viruses and accession numbers used in this study can be found in Table 1. Phylogenetic analysis of the spike RBD further reflected the close relatedness between Khosta -1 and -2 with BM48-31 and other clade 3 RBD viruses we have previously tested from Uganda and Rwanda [1,13] (Fig 1A). Clade 3 RBDs, including the Khosta viruses, contain a truncated surface-exposed loop, as compared to the ACE2-dependent, clade 1 viruses such as SARS-CoV, and additionally vary in many of the residues known for clade 1 viruses to interact with human ACE2 [1,2,13,14].

**Table 1:** Sarbecovirus sequences used in this study.

	Virus	Accession	Clade	Hostspecies	Location
1	SARSUrbani	AY278741	1	human	Guangdong,China(originofoutbreak)
2	WIV1	KF367457	1	Rhinolophussinicus	Yunnan,China
3	LYRa11	KF569996	1	Rhinolophusaffinis	Baoshan,Yunnan,China
4	Rs7327	KY417151	1	Rhinolophussinicus	Kunming,YunnanProvince,China
5	Rs4231	KY417146	1	Rhinolophussinicus	Kunming,YunnanProvince,China
6	Rs4084	KY417144	1	Rhinolophussinicus	Kunming,YunnanProvince,China
7	RsSHC014	KC881005	1	Rhinolophussinicus	Yunnan,China
8	SARS‐CoV‐2/Wuhan‐Hu‐1	MN908947	1	human	Wuhan,Hubei,China
9	SARS‐CoV‐2/B.1.1.529	OM212472	1	human	HongKong
10	RatG13	MN996532	1	Rhinolophusaffinis	Yunnan,China
11	RatG15	Guoetal.2021	4	Rhinolophusaffinis	MojiangCounty,YunnanPrvince,China
12	As6526	KY417142	2	Aselliscusstoliczkanus	Kunming,YunnanProvince,China
13	Yunnan2011	JX993988	2	Chaerephonplicata	Yunnan,China
14	Shaanxi2011	JX993987	2	Rhinolophuspusillus	Shaanxi,China
15	279‐2005	DQ648857	2	Rhinolophusmacrotis	Hubei,China
16	Rs4237	KY417147	2	Rhinolophussinicus	Kunming,YunnanProvince,China
17	Rs4081	KY417143	2	Rhinolophussinicus	Kunming,YunnanProvince,China
18	Rp3	DQ071615	2	Rhinolophuspearsoni	Nanning,Guangxi,China
19	Rs4247	KY417148	2	Rhinolophussinicus	Kunming,YunnanProvince,China
20	HKU3‐8	GQ153543	2	Rhinolophussinicus	Guangdong,China
21	HKU3-13	GQ153548	2	Rhinolophussinicus	Guangdong,China
22	GX2013	KJ473815	2	Rhinolophussinicus	Guangxi,China
23	Longquan‐140	KF294457	2	Rhinolophusmonoceros	Longquan,Zhejiang,China
24	YN2013	KJ473816	2	Rhinolophussinicus	Yunnan,China
25	Rf4092	KY417145	2	Rhinolophusferrumequinum	Kunming,YunnanProvince,China
26	ZXC21	MG772934	2	Rhinolophussinicus	ZhoushanCity,Zhejiang,China
27	ZC45	MG772933	2	Rhinolophussinicus	ZhoushanCity,Zhejiang,China
28	JL2012	KJ473811	2	Rhinolophusferrumequinum	Jilin,China
29	HuB2013	KJ473814	2	Rhinolophussinicus	Hubei,China
30	Rf1	DQ412042	2	Rhinolophusferrumequinum	Yichang,Hubei,China
31	HeB2013	KJ473812	2	Rhinolophusferrumequinum	Hubei,China
32	273‐2005	DQ648856	2	Rhinolophusferrumequinum	Hubei,China
33	BM48-31	NC014470	3	Rhinolophusblasii	StrandjaNaturePark,Bulgaira
34	Uganda	MT726044	3	Rhinolophusferrumequinum	Uganda
35	Rwanda	MT726045	3	Rhinolophusferrumequinum	Rwanda
36	Khosta1	MZ190137	3	Rhinolophusferrumequinum	GreaterCaucasus,Russia
37	Khosta2	MZ190138	3	Rhinolophushipposideros	GreaterCaucasus,Russia

### RBD from Khosta viruses mediate entry into human cells

Using our scalable sarbecovirus RBD entry platform, we replaced the RBD from SARS-CoV-1 spike with the Khosta RBDs and generated chimeric spike expression plasmids (Fig 1B) [1]. For comparison, we also included chimeric RBD spikes for other clade 3 RBDs we have previously tested (BM48-31, Uganda, Rwanda) as well as SARS-CoV-2 and related RaTG13 viruses. These chimeric spike expression constructs were used to produce BSL2-compatible viral reporter pseudotypes with Vesicular Stomatitis Virus expressing a dual GFP-luciferase reporter [1]. All of the chimeric spike proteins expressed to similar levels in mammalian cells and incorporated in Vesicular Stomatitis Virus (VSV). Chimeric spike with the RBD from BM48-31 and RaTG13 showed reduced incorporation but this did not correlate with viral entry phenotypes observed in later experiments (Fig 1C, 1D and 1E).

To test human cell compatibility, we first infected the human liver cell line, Huh-7, with pseudotypes bearing the chimeric Khosta RBD spikes. In the absence of the addition of an exogenous protease, trypsin, the pseudotypes exhibited almost no entry in these cells, which has been observed for other sarbecoviruses and is attributed to low endogenous expression of ACE2. However, when trypsin was included during the infection, entry signal strongly increased for SARS-CoV-1 and -2 RBDs as well as the Khosta RBDs (Fig 1D). As we and others have shown previously, trypsin enhancement of sarbecovirus entry is still receptor dependent, suggesting that the Khosta virus RBDs were using a receptor present in human cells to mediate infection [1,15].

### The RBD from Khosta 2 infects cells using human ACE2

To characterize potential receptors for the Khosta viruses, we performed a classic receptor tropism test, where we transfected Baby Hamster Kidney (BHK) cells with human orthologues of known coronavirus receptors and then infected with our pseudotype panel. Unlike 293T cells, which express low levels of human ACE2 and potentially other coronavirus receptors and have been shown to have low but measurable susceptibility to SARS-CoV infection, BHK cells are generally considered completely non-susceptible for sarbecoviruses unless a suitable receptor is supplemented [16]. The Khosta-1 RBD failed to infect cells expressing any of the human receptors, while Khosta 2 RBD clearly infected cells expressing human ACE2 (Fig 1E). The level of cell entry mediated by the Khosta 2 RBD was similar to RaTG13, a bat sarbecovirus with high similarity to SARS-CoV-2 in the RBD that also binds human ACE2, albeit with lower efficiency than the human pathogen [17,18] (Fig 1E). In contrast to the ACE2 results, only the human virus, HCoV-229E, could infect cells expressing Aminopeptidase N (APN), and MERS-CoV spike could only infect cells expressing dipeptidyl peptidase IV (DPP4)–the known receptors for these viruses (Fig 1E). While exogenous protease mediated Khosta-1 RBD entry into Huh-7 cells, addition of protease failed to facilitate Khosta-1 RBD entry in BHK cells transfected with various coronavirus receptors, suggesting trypsin-dependent Khosta-1 entry in Huh-7 cells may be independent of human ACE2 (Fig 1F).

To better compare entry efficiency between the viral spikes and human ACE2, we infected 293T cells that were stably transduced to over-express human ACE2 (Fig 1G). Because these cells ubiquitously express the receptor on their surface in high abundance, they are maximally susceptible to sarbecoviruses that can infect human ACE2. In agreement with a recent study exploring sarbecovirus RBD entry, both African clade 3 RBDs that we tested were also capable of using human ACE2, albeit with much lower efficiency [9] (Fig 1G).

### Khosta 2 may interface with ACE2 similar to other sarbecoviruses

**Fig 2:**
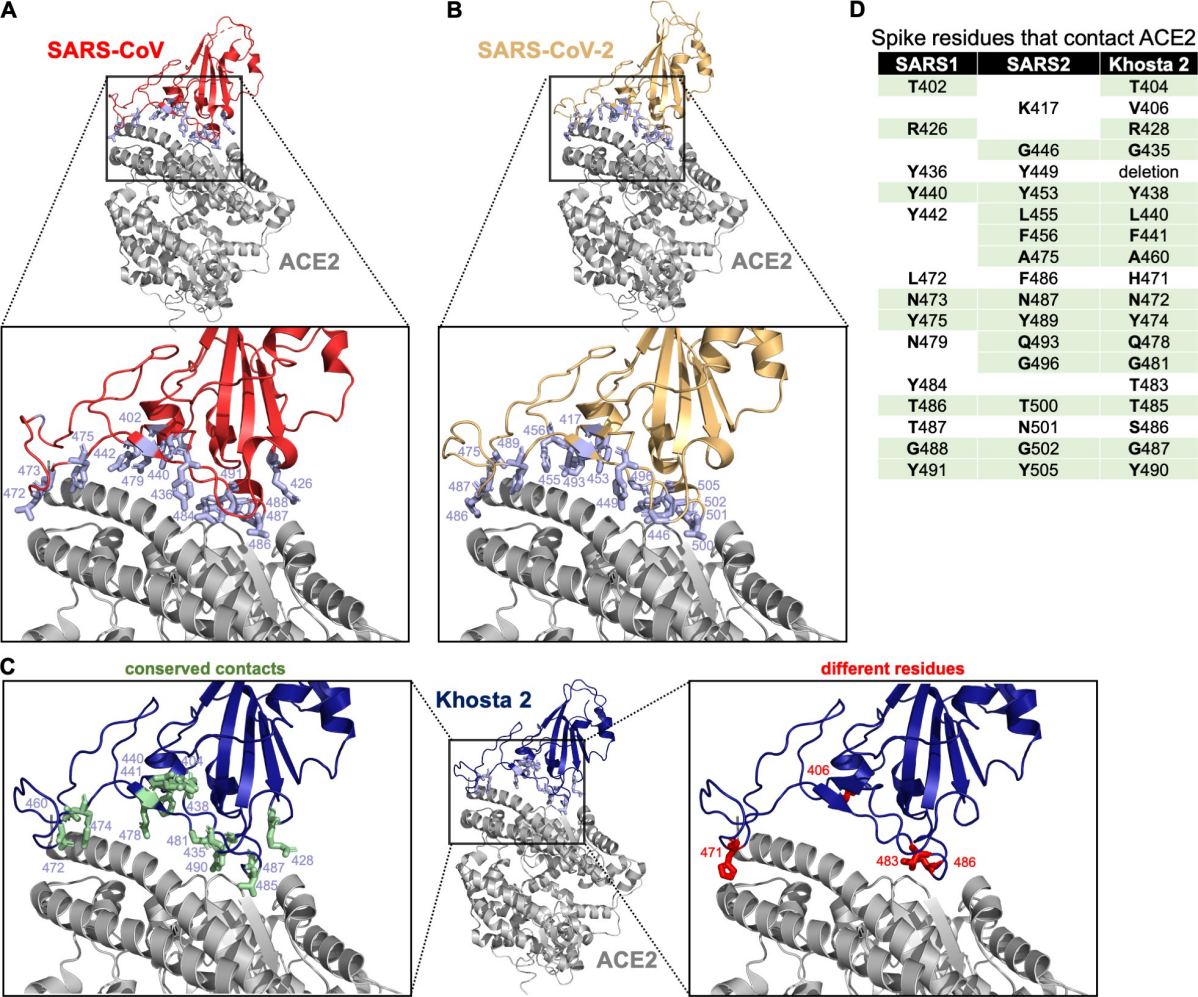
Modelled co-structure of Khosta 2 RBD and human ACE2. (**A**) Crystal structure of SARS-CoV RBD bound to human ACE2 (PDB ID: 2AJF) with contact points indicated in light blue. (**B**) Crystal structure of SARS-CoV-2 RBD bound to human ACE2 (PDB ID: 6M0J) with contact points indicated in light blue. (**C**) Predicted structure of Khosta 2 RBD bound to human ACE2 with contact points identical to either SARS-CoV or SARS-CoV-2 spike indicated in light green and resides that are different indicated in red. (**D**) ACE2-contact point comparison between SARS-CoV, SARS-CoV-2 and Khosta 2 spike. Residues that are identical between Khosta 2 and SARS-CoV or SARS-CoV-2 are shaded in light green.

We wondered how the protein interaction between Khosta 2 and human ACE2 compared with other known clade 1 RBDs for which structural data is available (Fig 2). We had the structure for the Khosta 2 RBD predicted from published structural data, which we subsequently aligned to the co-structures for SARS-CoV and SARS-CoV-2 bound to ACE2 (Fig 2A–2C). Many of the residues in these two clade 1 RBDs were conserved in the Khosta 2 RBD (Fig 2D), which likely contribute toward the interface between Khosta 2 RBD and human ACE2 (Fig 2C) [19,20].

### Full-length Khosta spikes infect human cells through ACE2

**Fig 3:**
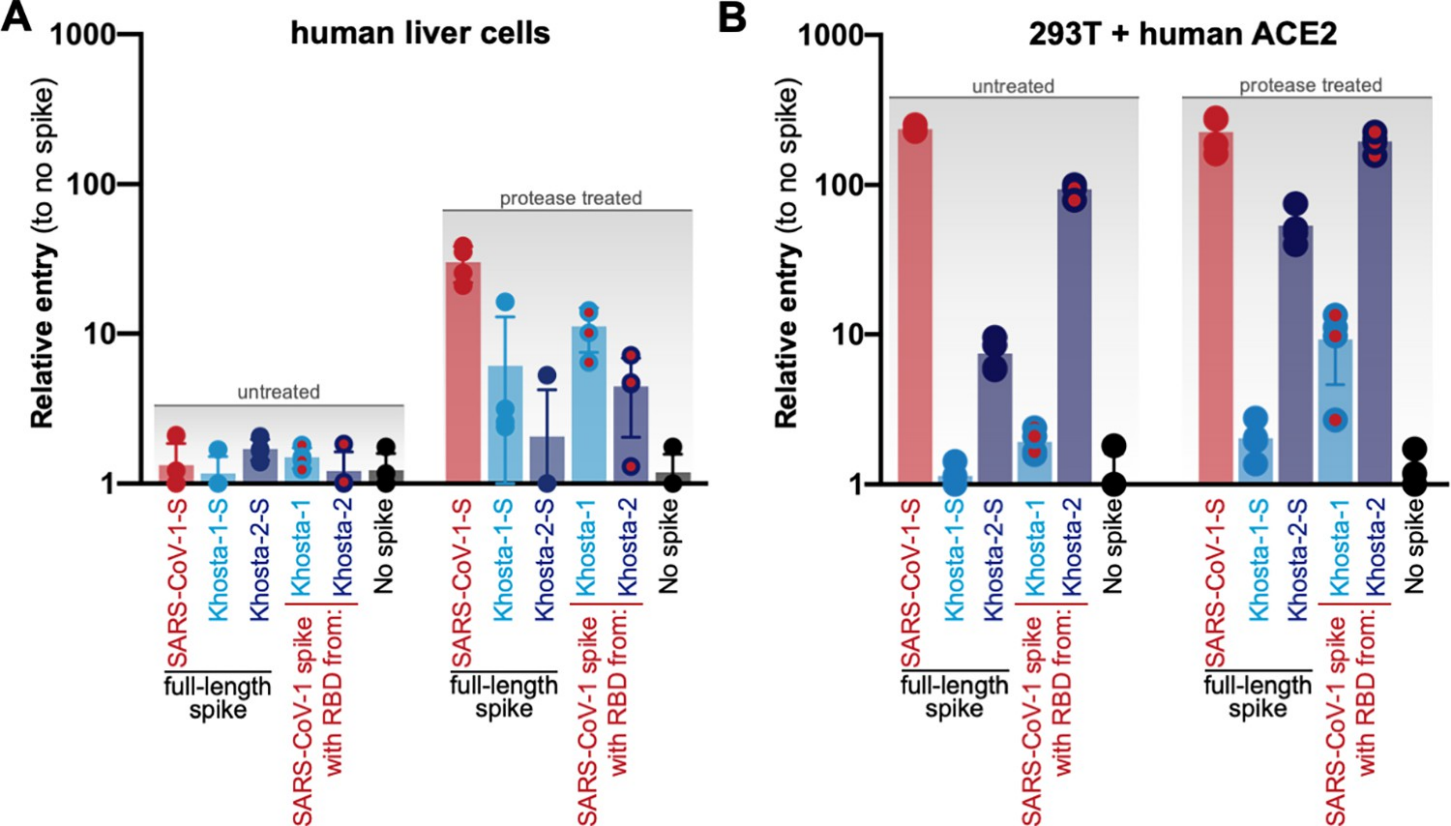
Full-length Khosta spikes infect human cells. (**A**) Huh-7 or (**B**) 293T cells that stably express human ACE2 cells were infected with VSV pseudotypes bearing the indicated spike protein.

While the RBD from Khosta 2 can use human ACE2 in functional assays (Fig 1) and bind ACE2 as a purified protein fragment [10], other domains in spike vary between the Khosta and SARS-CoV spikes. We had the full-length Khosta spike genes synthesized, generated viral pseudotypes and tested their infectivity on human cells (Fig 3). Similar to the chimeric SARS-CoV-based spikes, full-length Khsota spikes could also infect Huh-7 cells in the presence of trypsin (Fig 3A) and the Khosta 2 spike was capable of infecting 293T cells expressing human ACE2 even in the absence of trypsin (Fig 3B). Analagous to what we have shown with other full-length Sarbecovirus spikes, the full-length Khosta 2 spike was less infectious than the chimeric SARS-CoV-based spike (Fig 3B) [1].

### A SARS-CoV-2 based, Khosta2-chimeric spike is resistant to serum from SARS-CoV-2 vaccinated individuals

**Fig 4:**
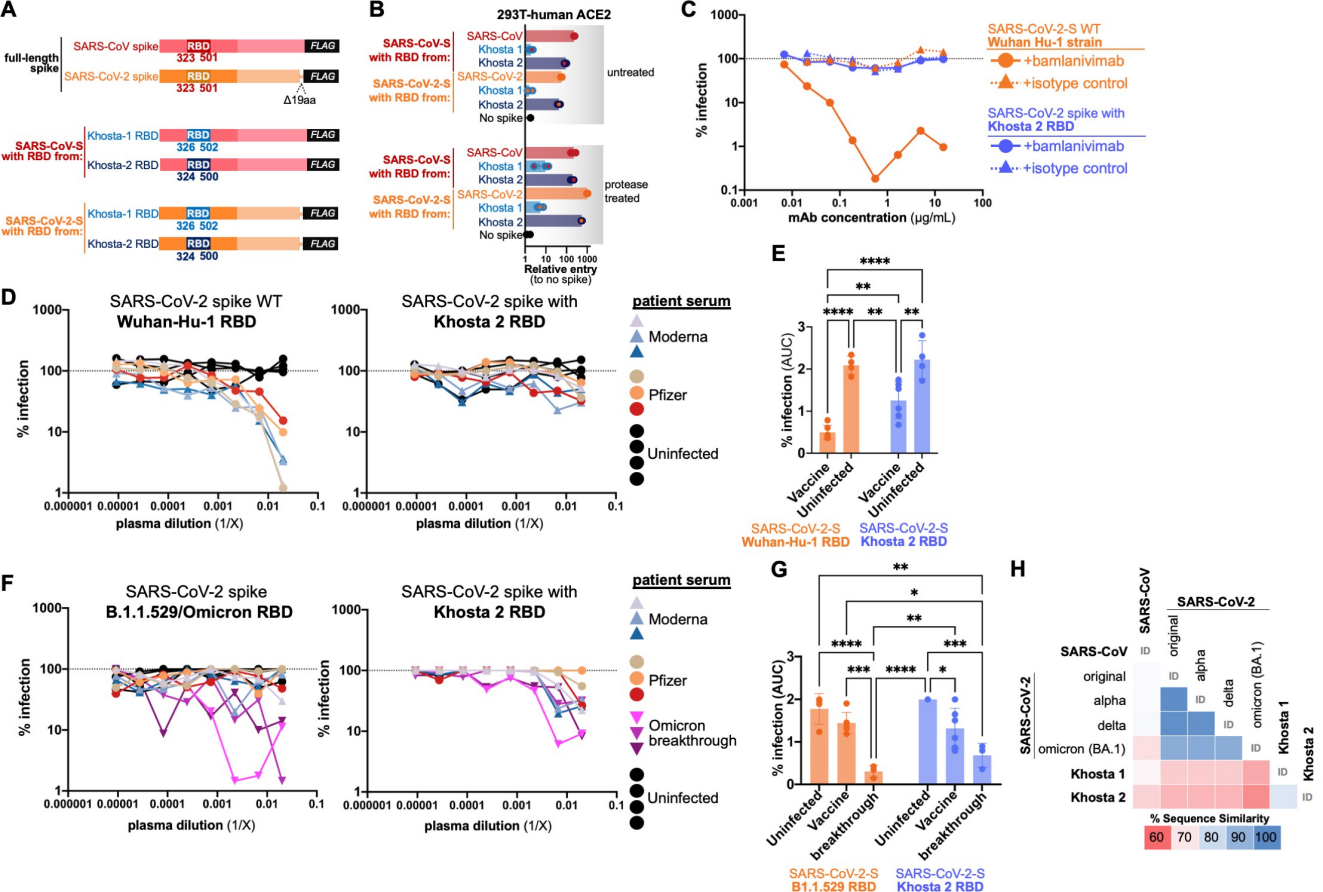
Chimeric SARS-CoV-2-Khosta 2 spike is resistant to current vaccines. (**A**) The RBD from SARS-Cov-2 spike was replaced with Khosta 2 RBD. (**B**) Pseudotpyes with indicated chimeric spikes were used to infect 293T cells stably expressing human ACE2. Pseudotypes were combined with (**C**) bamlanivimab or (**D**) vaccinated patient serum at various concentrations and used to infect 293T-hACE2 cells.(**E**) Area under the curve analysis for data in panel D. p-value 0.0021 (**), 0.0002 (***), <0.0001 (****). (**F**) Pseudotypes with Khosta 2 or Omicron variant RBD were combined with serum from vaccinated patients with breakthrough Omicron infection and used to infect 293T-hACE2 cells. (**G**) Area under the curve analysis for data in panel F. p-value 0.0021 (**), 0.0002 (***), <0.0001 (****) (**H**) Sequence identity matrix for RBD amino acid sequences from Khosta 2 and known SARS-CoV-2 variants of concern.

SARS-CoV-2 can infect a wide range of species and has now spilled back into both wild and domestic animals [21–30]. Many animal species carry their own coronaviruses, and with the discovery of additional ACE2-dependent sarbecoviruses in broader geographic regions, the risk of new recombinant viruses is rising. To mimic the potential recombinant threat from the Khosta viruses, we generated VSV pseudotyped particles carrying a chimeric SARS-CoV-2-based spike with the RBD from the Khosta viruses (Fig 4A). Similar to our earlier SARS-CoV-based spikes, the SARS-CoV-2 chimeric spikes were also infectious in 293T cells expressing human ACE2 (Fig 4B). To assess if the ACE2-dependent Khosta 2 RBD and SARS-CoV-2 RBD were cross-reactive, we incubated pseudotyped particles with increasing amounts of the SARS-CoV-2 RBD-specific monoclonal antibody, Bamlanivimab. Surprisingly, while SARS-CoV-2 spike was effectively neutralized by the antibody, the SARS-CoV-2 spike with the Khosta 2 RBD was completely resistant, suggesting little cross-reactivity between the two RBDs (Fig 4C). We repeated the pseudotype experiment using serum from vaccinated individuals and saw a similar trend: the wild-type SARS-CoV-2 spike was easily inhibited by serum from individuals who received 2 doses of either the Moderna or Pfizer vaccine, but the SARS-CoV-2-Khosta 2 RBD spike was resistant (Fig 4D). At higher dilutions of serum, there was a reduction in the chimeric spike infectivity, but this was significantly less than the wildtype spike at similar serum concentrations (Fig 4E). As individuals receive additional SARS-CoV-2 vaccine boosters and SARS-CoV-2 continues to circulate, new viral variants emerge that can alter and broaden the immune response to sarbecoviruses [31, 32]. To see how this reduction in neutralization compares between Khosta 2 and SARS-CoV-2 circulating variants of concern, we generated and tested a SARS-CoV-2 spike with the RBD from an Omicron strain (B.1.1.529) against serum from vaccinated individuals with Omicron breakthrough (Fig 4F and 4G). While SARS-CoV-2 spike with the micron-RBD was effectively neutralized by the patient serum, the Khosta 2 RBD was still slightly resistant even at higher serum concentrations (Fig 4F and 4G). The Khosta 2 RBD shares approximately 60% similarity with various SARS-CoV-2 spikes at the amino acid level, which likely underlies its low cross-reactivity (Fig 4H). Taken together, these results demonstrate that new recombinant sarbecoviruses may pose a threat to current SARS-CoV-2 vaccines.

## Discussion

Khosta 1 and 2 viruses are most closely related to other clade 3 RBD viruses, which have been found across a much wider geographic range than the clade 1 viruses [1,13,33,34]. As the researchers who initially discovered the Khosta viruses note with their findings: the Khosta bat sarbecoviruses are genetically distinct from human SARS-CoVs in that they lack genetic information encoding for some of the genes thought to antagonize the immune system and contribute to pathogenicity, such as Orf8 [12]. Unfortunately, because coronaviruses are known to recombine in co-infected hosts, the recent identification of SARS-CoV-2 spillover from humans back in wildlife populations opens the possibility of new human-compatible sarbecoviruses [21–23,28–30].

In the presence of trypsin, both Khosta-1 and -2 RBDs and spike were capable of infecting human cells, with Khosta-1 performing notably stronger than Khosta 2, however in our receptor-specific assays, only Khosta 2 could infect cells expressing human ACE2 without exogenous protease (Figs 1D–1G and 3). In contrast to our earlier results with African clade 3 sarbecovirues on receptor-transfected BHKs, both the Uganda and Rwanda viruses were able to utilize human ACE2 in cells stably overexpressing the receptor [13] (Fig 1G). The approximate 10-fold entry signal measured in our assay is identical in strength to recent findings reporting that the Uganda virus RBD could use human ACE2 [9]. Thus, while BHK cells transfected with host receptors represent an effective method to distinguish the obvious receptor preference for coronaviruses, transduced human cell lines allow for improved detection of low-affinity interactions. Taken together, these findings from our study and others demonstrate low level human ACE2 usage across the RBD clade 3 sarbecoviruses.

A recent study has demonstrated a single point mutation, T498W, can be introduced into some clade 3 sarbecovirus spike RBDs, including Khosta 1, that broadens viral species tropism from bat to human ACE2 [10]. Curiously, we observed that while Khosta 1 spike was capable of infecting human cells in the presence of protease, Khosta 1 RBD failed to efficiently transduce cells over-expressing human ACE2 (Figs 1F, 1G and 3). While both our study and the previous one demonstrate wildtype Khosta 1 RBD cannot use human ACE2 efficiently, our data showing robust Khosta 1 entry into Huh-7 cells suggests an additional entry mechanism into human cells may be available to at least some clade 3 RBDs (Fig 1A, 1D and 1F). We have previously shown that a small number of clade 2 RBDs, such as As6526 and Rs4081, also exhibit protease-mediated, ACE2-independent entry, and similar phenotypes have been described for other bat coronaviruses [1,35]. Analogous to Khosta 1, the completely ACE2-independent RBD clade 2 sarbecovirus, Rs4081, also efficiently infects Huh-7 cells in the presence of trypsin [36]. Because not all of the RBD clade 2 and 3 sarbecoviruses exhibit trypsin-dependent entry in our comparative assays with chimeric spikes, these findings collectively suggest that some coronaviruses may infect human cells through a presently unknown receptor. Sarbecoviruses have been shown to co-circulate in bats, so this variation in receptor usage among closely related viruses may even represent an evolutionary strategy for viral persistence within the reservoir host population [2].

Current universal sarbecovirus vaccines in development include mostly clade 1 viruses and one of the clade 2 viruses but do not include any members from clade 3 [37,38]. Our results suggest there is little cross-reactivity between clade 1 and clade 3 RBDs that use human ACE2, even though their interactions are likely very similar (Figs 2 and 4C). More concerning was our observation that serum from vaccinated individuals was less effective at neutralizing pseudotypes when just the SARS-CoV-2 RBD was replaced with the Khosta 2 RBD (Fig 4D–4G). These findings are not too surprising given that the Khosta 2 RBD only shares about 60% sequence identity with SARS-CoV-2, and the neutralizing antibodies elicited by the vaccines from Moderna or Pfizer are directed primarily toward the RBD [39] (Fig 4E and 4F). Bamlanivimab makes contact with 17 residues on SARS-CoV-2 Wuhan strain, and Khosta2 shares only 10 or the 17 residues. Moreover, loss of bamlanivimab binding has been mapped to E484A and Q493R in Omicron [40,41]; Khosta2 encodes a G435 at a position analogous to E484 on SARS-CoV-2, providing a possible basis for escape from bamlanivimab. Curiously, the Khosta 2 RBD is least similar to the currently circulating Omicron variant of SARS-CoV-2; with each new variant of concern decreasing in similarity to Khosta 2 (Fig 4H). Given that natural infection or vaccination with a whole spike raises antibodies directed at other regions of spike, it is still possible that new sarbecoviruses or recombinant SARS-CoV-2 would be neutralized by serum from some individuals.

Our findings with chimeric, SARS-CoV-2 spike show that just replacing the RBD is sufficient to reduce efficacy of SARS-CoV-2 spike-directed vaccines (Fig 4). However, sarbecovirus recombination in nature typically occurs via template switching resulting in acquisition of regions larger than the NTD [42]. Thus, a naturally recombinant virus with Khosta 2 may actually acquire more Khosta 2 spike, which as we show here with full protein, is also infectious against human cells and ACE2 (Fig 3). Taken together, our findings with the Khosta viruses underscore the urgent need to develop broader-protecting universal Sarbecovirus vaccines.

## Methods

### Ethics statement

Deidentified plasma samples were from subjects recruited from the Greater New Orleans community under Tulane Biomedical Institutional Review Board (federal wide assurance number FWA00002055, under study number 2020–585).

### Phylogenetic analysis

Genbank accession numbers for all sarbecovirus spike sequences used in this study are available in Table 1. Amino acid sequences for the receptor binding domain of the spike glycoprotein were aligned using ClustalW multiple sequence alignment with default parameters. A maximum likelihood phylogenetic tree was inferred with PhyML v. 3.0 [43] using the ‘WAG’ matrix +G model of amino acid substitution as selected by Smart Model Selection method with 1000 bootstrap replicates [44]. The final tree was then visualized as a cladogram with FigTree v1.4.4 (https://github.com/rambaut/figtree).

### Plasmids and sequences

Untagged human orthologues of ACE2 (Q9BYF1.2), APN (NP_001141.2), and DPP4 (XM_005246371.3) were described previously [1]. Spike sequences from HCoV-229E (AB691763.1), MERS-CoV (JX869059.2), and SARS-CoV-1 (AY278741) were codon-optimized, appended with a carboxy-terminal FLAG tag sequence separated by a flexible poly-glycine linker and cloned into pcDNA3.1+ as previously described [1]. SARS-CoV-2 spike (MN997409.1) was codon optimized, modified to including silent cloning sites flanking the RBD, and C-terminal 19 amino acid truncation was introduced to enhance pseudotyping [1,45]. The SARS-CoV-1 RBD was removed with KpnI and XhoI, and the SARS-CoV-2 RBD was removed with BamHI and PflMI. Codon-optimized, synthesized RBD fragments were cloned into the spike backbones as previously described [1]. Accession numbers for all spikes sequences in this study can be found in Table 1. RatG15 sequence used for phylogenetic analysis was direct from the publication [11]. Plasmid sequences were verified either by standard Sanger methods (Azenta Life Sciences), or Oxford Nanopore-based full-plasmid sequencing (Plasmidsaurus).

### Cells and pseudotype assay

293T, 293T-hACE2 stable cells, Huh-7 (human liver cells), and BHKs were maintained under standard cell culture conditions in DMEM with L-glutamine, antibiotics, and 10% Fetal Bovine Serum. Single-cycle, Vesicular Stomatitis Virus (VSV) pseudotype assays were performed as previously described [1]. Briefly, 293T “producer cells” were transfected with spike plasmids or empty vector as a “no spike” control and infected 24-hours later with VSV-ΔG-luc/GFP particles, generating chimeric-spike pseudotyped particles that were harvested 72 hours post-transfection and stored at -80°C. Target cells were plated in 96-well format, and spin-infected in quadruplicate with equivalent volumes of viral pseudotypes at 1200xG for 1 hour at 4°C. Infected cells were incubated for approximately 18–20 hours and luciferase was measured using the Promega BrightGlo luciferase kit following manufacturers’ instructions (Promega). Entry signal was normalized to the average signal for the “no spike” control. Plates were measured and analyzed in triplicate. Data are representative of four complete biological replicates. All graphed data points in this study are available in S1 Data.

### Western blot

Viral pseudotypes were concentrated and 293T producer cells were lysed in 1% SDS and clarified as described previously [1]. Lysates were analyzed on 10% Bis-Tris PAGE-gel (Thermo Fisher Scientific) and probed for FLAG (Sigma-Aldrich; A8592; 1:10000); GAPDH (Sigma-Aldrich, G8795, 1:10000); and/or VSV-M (Kerafast, 23H12, 1:5000). Signal was detected using SuperSignal West substrate (Thermo-Fisher).

### Protein structural comparison

The structures for SARS-CoV bound to human ACE2 (PDB ID: 2AJF), or SARS-CoV-2 bound to human ACE2 (PDB ID: 6MOJ) were aligned in SwissPDB Viewer. Khosta 2 RBD structure was predicted using SwissModel using PDB ID: 7SBK as the template structure. Structures were visualized in PyMol v.2.4.0.

### Patient serum samples

Sera from six vaccinated (3 Pfizer and 3 Moderna), 4 uninfected donors, and three vaccinated individuals with breakthrough infection (presumed Omicron based on infection dates between late December 2021 and end of January 2022) were kindly provided by Tulane University. Patient samples were collected in 2021–2022 from individuals who had received two vaccine doses and/or breakthrough infection.

### Monoclonal and serum neutralization assays

293T-ACE2 cells were seeded at 2.5 x 10^4^ cells/well in 96-well format and grown for 24h. Pseudotyped virus particles were titered on 293T-ACE2 cells by limiting dilution as previously described [46]. Pseudotypes were diluted to 500 focus-forming units and then incubated with the sera (1:3 diluted from 1:50) at 37°C. Ultra-LEAF IgG1 isotype (Biolegend) and Bamlanivimab (Lilly) were as negative and positive controls (started with 15ug/ml with 3x dilution). After 1h incubation, 293T-ACE2 cells were inoculated with the virus-antibody mixtures and centrifuged at 1200xG for 1 hour at 4°C. Cells were transferred back to the incubator and luciferase was measured at 24 hours post-infection (Bright-Glo Luciferase Assay System, Promega). Values were normalized to those derived from wells with pseudovirus but without sera (100% infection). Data were input and analyzed by Prism 9. AUC of percentage of infection from each vaccinated and infected were counted and the difference of significance were analyzed by one-way ANOVA test.

## Supporting information

S1 Data Excel file with all graphed data points from main text figures. (XLSX)
